# Increased blood *COASY* DNA methylation levels a potential biomarker for early pathology of Alzheimer’s disease

**DOI:** 10.1038/s41598-020-69248-9

**Published:** 2020-07-22

**Authors:** Nobuyuki Kobayashi, Shunichiro Shinagawa, Hidehito Niimura, Hisashi Kida, Tomoyuki Nagata, Kenji Tagai, Kazuya Shimada, Naomi Oka, Ryo Shikimoto, Yoshihiro Noda, Shinichiro Nakajima, Masaru Mimura, Masahiro Shigeta, Kazuhiro Kondo

**Affiliations:** 1 Department of Virology, The Jikei University School of Medicine, Tokyo, Japan; 2 Department of Psychiatry, The Jikei University School of Medicine, Tokyo, Japan; 3 Department of Neuropsychiatry, Keio University School of Medicine, Tokyo, Japan

**Keywords:** Alzheimer's disease, Predictive markers, DNA methylation

## Abstract

Early diagnosis of dementia including Alzheimer’s disease (AD) is an urgent medical and welfare issue. However, to date, no simple biometrics have been available. We reported that blood DNA methylation levels of the *COASY* gene, which encodes coenzyme A synthase, were increased in individuals with AD and amnestic mild cognitive impairment (aMCI). The present study sought to replicate these findings with larger numbers of samples. Another objective was to clarify whether *COASY* methylation is associated with neurodegeneration through a comparison of AD, AD with cardiovascular disease (CVD), and vascular dementia (VaD). We measured blood *COASY* methylation levels in normal controls (NCs) (*n* = 200), and individuals with aMCI (*n* = 22), AD (*n* = 151), and VaD (*n* = 21). Compared with NCs, they were significantly higher in individuals with aMCI and AD. Further, they were significantly higher in AD patients without cardiovascular diseases compared to AD patients with them. These findings suggest that *COASY* methylation levels may be related to neurodegeneration in AD.

## Introduction

In recent years, the increased incidence of dementia has become a social problem for the aging of society and aging is a major risk factor for dementia. Alzheimer’s disease (AD)^1^ is the most common type of dementia, followed by vascular dementia (VaD)^2^. AD is a neurodegenerative disease characterized by progressive neuronal loss with deposition of abnormal proteins. The neurodegenerative disease goes through the early stage called mild cognitive impairment (MCI) and ultimately develops into dementia. On the other hand, in the case of VaD, cognitive impairment is caused by cerebrovascular disease (CVD). Differential diagnosis in the early stage of dementia is crucial because the therapeutic strategy depends on the type of dementia.

Neuropathological changes in AD begin with the deposition of amyloid β-protein (Aβ), followed by the deposition of phosphorylated tau protein^1,3^. These changes precede cognitive impairment. For the early diagnosis of AD, amyloid positron emission tomography (PET) and tau PET are useful but these modalities are costly and difficult to use in all individuals in the clinical setting. Although decreases in Aβ_42_ and increases in tau protein in cerebrospinal fluid are useful biomarkers^3^, measurements are invasive and insufficient for differential diagnosis at an early stage of AD. Therefore, viable and less invasive methods are required in the context of feasibility. Interacting with various environmental factors, DNA methylation, a known epigenetic mechanism, affects gene expression and phenotypes without any changes in gene sequences^4^. Since DNA methylation changes with aging^5,6^ blood DNA methylation levels could be a potential diagnostic biomarker for dementia^7^.

The *COASY* gene encodes coenzyme A (CoA) synthase and it is involved in the biosynthesis of CoA from pantothenic acid. CoA synthase is mainly present in the mitochondrial matrix and mutations in this gene alter enzymatic activity. It has been reported that there is an association between mutations in *COASY* and neurodegeneration with brain iron accumulation (NBIA)^8^ and that a single-nucleotide polymorphism of *COASY* exon 4 is a risk factor for early onset of AD in females with Down syndrome^9^. Moreover, via hyper-acetylation of kinases associated with mitosis, *COASY* knock-down causes prolonged mitosis and failure of cytokinesis^10^. Thus, the aforementioned findings suggest that *COASY* may play an important role in neurodegeneration. In a previous pilot study using methylation-sensitive high-resolution melting (MS-HRM) analysis^11^, we reported that DNA methylation in the *COASY* gene promoter region is increased in individuals with amnestic MCI (aMCI) (*n* = 28) and AD^12,13^ (*n* = 30) in comparison with normal controls (NCs) (*n* = 30), suggesting that DNA methylation in the *COASY* gene promoter region could be a diagnostic marker for aMCI and AD.

However, it still remains unclear what roles *COASY* plays in neurodegeneration, and VaD, a disease differing from AD. Moreover, changes in *COASY* DNA methylation in the brain remain unclear because it would be necessary to measure them through postmortem examination of the brains of individuals with AD and NCs. Therefore, the aim of the present study was to expand the scope of our previous study and investigate changes of *COASY* DNA methylation in individuals with AD, VaD, and NCs. Through these experiments, we further aimed to demonstrate that changes in *COASY* methylation levels are associated with the upstream cascade of the pathophysiology of AD and could be a useful blood biomarker for the diagnosis of AD.

## Materials and methods

### Subjects

Individuals in the present study were independent of those in our previous study^12,13^. Individuals with aMCI, AD, and VaD were enrolled from the memory clinics of the following: the Jikei University Hospital, Tokyo (*n* = 51); the Jikei University Kashiwa Hospital, Kashiwa City, Chiba Prefecture (*n* = 80)^14^; and Aira-no-mori Hospital, Aira-gun, Kagoshima Prefecture (*n* = 63). Diagnosis of aMCI was determined by the criteria defined by Peterson^15^ and included both aMCI-single domain- and MCI-multiple domain-type individuals. AD was diagnosed based on the US National Institute of Neurological and Communicative Disorders and Stroke and the Alzheimer's Disease and Related Disorders Association (NINCDS-ADRDA) criteria^16^. The AD group was further stratified for presence of ischemic changes (hereafter, this category referred to as AD with CVD subgroup)^17^. All individuals with AD underwent 1.5 T magnetic resonance imaging (MRI) including T1 weighted images (T1WI), T2 weighted images (T2WI), and fluid attenuated inversion recovery (FLAIR). AD with CVD was defined as having, in addition to AD, cerebrovascular lesions such as lacunar infarctions, white matter lesions (WMLs), and microbleeding on MRI by expert neuropsychiatrists. VaD was diagnosed according to the criteria of the National Institute of Neurological Disorders and Stroke and Association Internationale pour la Recherche et l’Enseignement en Neurosciences (NINDS-AIREN)^2,18^. The exclusion criteria for patients included (1) being 91 years and older, (2) having been diagnosed with severe mental illness, or (3) having history of head trauma or substance use disorders.

NCs were recruited from inhabitants of Arakawa Ward in the eastern Tokyo metropolitan area as a part of the Arakawa 65 + Study and Japan Prospective Studies Collaboration for Aging and Dementia (JPSC-AD; https://www.eph.med.kyushu-u.ac.jp/jpsc/en/), which was a prospective cohort study that aimed to explore healthy longevity in elderly individuals between 65 and 84 years of age^19^. From the individuals (*n* = 1,099) recruited for the research, 200 were selected at random and enrolled in the present study. NCs had normal cognitive function and did not meet the clinical criteria for any types of dementia or MCI.

The Mini-Mental State Examination (MMSE) was administered to all of the patient groups by expert clinical psychologists^20^.

### APOE genotyping

Genomic DNA was extracted from peripheral blood cells using a standard method^14^. *APOE* genotypes (rs429358 and rs7412) were determined by allelic discrimination on an Applied Biosystems 7,300 real-time PCR System (Thermo Fisher Scientific). The amplifications were performed in duplicate in a total volume of 25 µL containing 12.5 µL of 2x TaqMan Genotyping Master Mix (Thermo Fisher Scientific), 0.625 µL of 40 × Primer and TaqMan Probe dye mix (assay ID C—3084793_20 or C—904973_10) (Thermo Fisher Scientific), 0.2 µL of the genomic DNA, and 11.675 µL of PCR-grade water. The thermal profile was 95 °C for 10 min, followed by 40 cycles of 95 °C for 15 s and 60 °C for 1 min. Data analysis used Sequence Detection Software version 1.4 (Thermo Fisher Scientific).

### Un-methylated DNA and methylated DNA

The unmethylated human genomic DNA used was EpiScope Unmethylated HCT116 DKO gDNA (Takara Bio), DNA derived from double knock-out HCT116 cells (*DNMT1* and *DNMT3B* knocked out). The methylated human genomic DNA was EpiScope Methylated HCT116 gDNA (Takara Bio), made by high-degree methylation of un-methylated DNA using CpG methylase.

2 µg of each genomic DNA sample was bisulfite-converted using an EpiTect Plus DNA Bisulfite Kit (Qiagen) and purified. Various mixtures of bisulfited unmethylated DNA and bisulfited methylated DNA were made to produce a calibration curve for methylation rates of 100%, 75%, 50%, 25%, 5%, and 0%.

### Methylation-sensitive high-resolution melting (MS-HRM) analysis

500 ng–1 µg of each genomic DNA sample was bisulfite-converted using an EpiTect Plus DNA Bisulfite Kit (Qiagen). Samples were bisulfited at the same concentration as for the bisulfited control DNA used for the calibration curve, and then purified.

**Figure 1:**
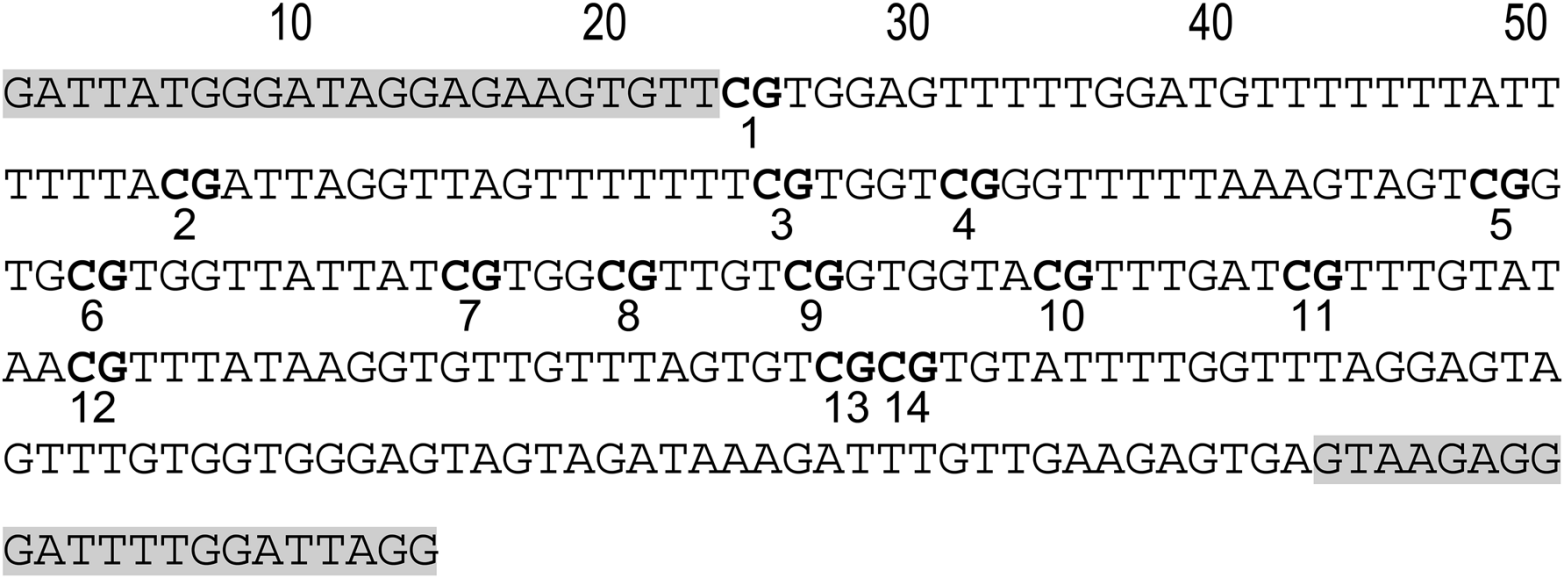
PCR amplification region. This shows the PCR amplification sequences of the bisulfited human *COASY* region (**A**) and the bisulfited mouse *COASY* region (**B**). The gray areas indicate the loci of primers.

**Figure 2:**
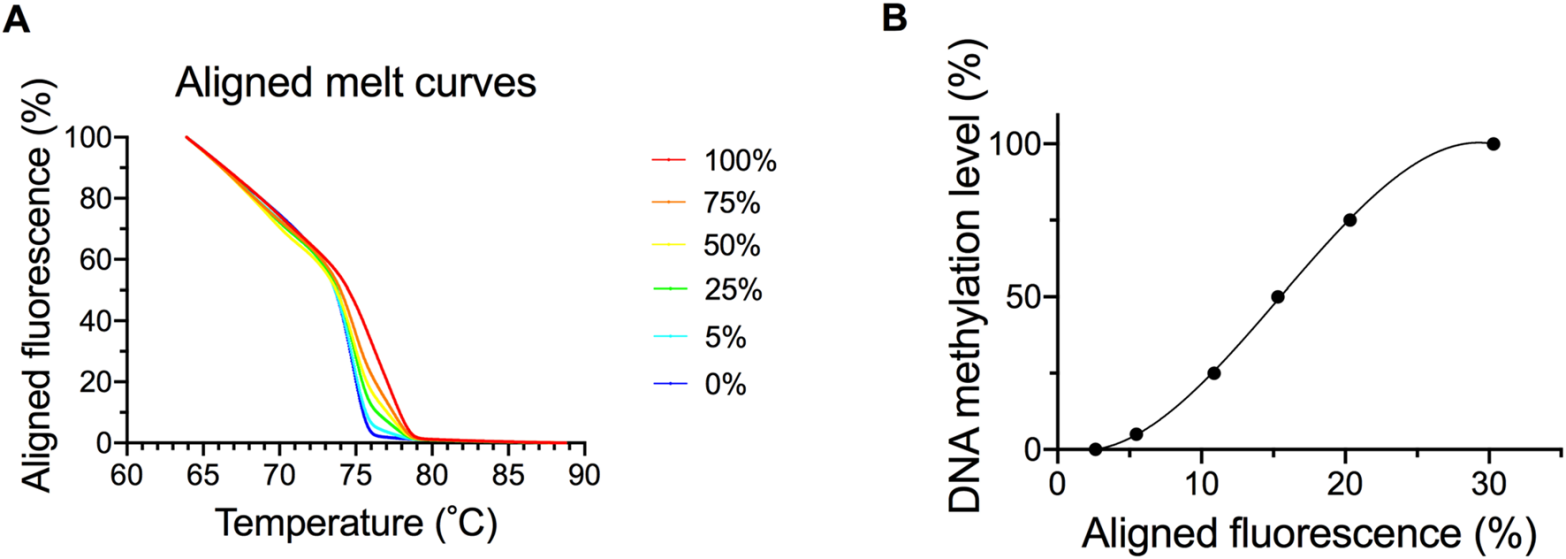
Aligned melt curves and calibration curve. This shows aligned melt curves (**A**) and the calibration curve (**B**) used in MS-HRM analysis for bisulfited DNA. 100%, 75%, 50% 25%, 5% and 0% (control) bisulfited DNA are indicated with red, orange, yellow, green, light blue and blue lines, respectively.

The primers for the human *COASY* promoter region were the same as those used in our previous research^13^. Regarding the procedure, we designed primers with Methyl Primer Express Software v1.0 (Thermo Fisher Scientific) in a region including the sequences of the probe (Target ID cg01756799) used with the Illumina Infinitum HD Methylation Assay within Homo sapiens *COASY*, transcript variant 1, mRNA and Genomic Sequence (GenBank accession number NM_025233, Gene ID: 80,347). We made fine adjustments to the sequences manually. Amplifications were performed in a total volume of 20 µL containing 10 µL of 2x MeltDoctor HRM Master Mix (Applied Biosystems), 0.12 µL of 50 µM forward primer, 0.12 µL of 50 µM reverse primer, 0.6 µL of the bisulfited DNA, and 9.16 µL of PCR-grade water. The thermal profile was 95 °C for 10 min, 50 cycles of 95 °C for 15 s, and 60 °C for 60 s. The primers were as follows: human *COASY* forward primer, 5′-GATTATGGGATAGGAGAAGTGTT-3′, human *COASY* reverse primer, 5′-CCTAATCCAAAATCCCTCTTAC-3′. The amplification size was 264 bp and included 14 CpG sites (Fig. 1). MS-HRM was performed with the Applied Biosystems QuantStudio 12 K Flex Real-Time PCR System (Thermo Fisher Scientific). Using the bisulfited control DNA values, the calibration curve was obtained by regression using a cubic function and DNA methylation levels of samples were quantified. The aligned melt curves and calibration curve are shown in Fig. 2A,B.

### Statistical analysis

The Shapiro–Wilk test was used to assess the normality of data. Multiple-group comparisons of non-parametric data were conducted using the Kruskal–Wallis test and the Dunn–Bonferroni correction was used for subsequent testing as needed. Sex and *APOE* genotype were compared using the *chi*-squared test. Spearman's rank correlation coefficients were used to investigate correlations between individual demographic characteristics and DNA methylation levels. Multiple linear regression analysis was then conducted with *COASY* DNA methylation levels in the AD group as the dependent variable, and age, sex, and MMSE score, for which differences (*P*-values less than 0.1) were noted using Spearman’s rank correlation coefficients, as forced entry variables. The Mann–Whitney U test was used for two-group comparisons. *P* < 0.05 was considered statistically significant.

Statistical analysis was conducted using SPSS Statistics 21.0 for Windows (IBM) and Prism 8 for macOS (GraphPad Software).

### Ethics statement

The study was approved by the Ethics Committees of the Jikei University School of Medicine and Keio University School of Medicine and written informed consent was obtained from all individuals. For participants whose capacity to consent was compromised, caregivers who were the spouse or a relative within the second degree consented on their behalf as a substitute decision-maker only when they provided assent for the participation. Our present study was performed in accordance with the principles of the Declaration of Helsinki and the Ethical Guidelines for Medical and Health Research Involving Human Subjects in Japan.

## Results

### Participant characteristics

Participant characteristics are presented in Table 1. The AD group consisted of 115 individuals diagnosed as AD without CVD and 36 individuals diagnosed as AD with CVD.

**Table 1:** Participants characteristics.

	NC	aMCI	AD	VaD	*P* values of main statistics
*n*	200	22	151	21	
Age (years) mean ± SEM (min–max)	76.6 ± 0.3 (66–86)	78.7 ± 0.5 (76–83)	81.1 ± 0.5 (59–90)	81.9 ± 1.7 (59–89)	< 0.0001
Female: male (%)	60.0: 40.0	59.1: 40.9	72.2: 27.8	38.1: 61.9	0.007
Duration of disease (years) mean ± S.E.M. (min–max)	–	1.68 ± 0.30 (0.33–6.00)	2.99 ± 0.17 (0.08–10.0)	2.10 ± 0.61 (0.08–11.0)	–
Age at onset (years) mean ± S.E.M. (min–max)	–	76.9 ± 0.6 (70–82)	77.9 ± 0.6 (53–89)	79.4 ± 1.7 (58–88)	–
Education (years) mean ± S.E.M. (min–max)	12.0 ± 0.2 (2–22)	11.6 ± 0.7 (6–16)	10.9 ± 0.2 (0–21)	9.5 ± 0.5 (6–14)	< 0.0001
MMSE score mean ± S.E.M. (min–max)	27.9 ± 0.1 (22–30)	26.5 ± 0.4 (23–30)	17.3 ± 0.5 (0–28)	9.8 ± 1.7 (0–26)	< 0.0001
*APOE* ε2/ε2 no. (%)	0 (0.00)	1 (4.55)	0 (0.00)	0 (0.00)	–
*APOE* ε2/ε3 no. (%)	12 (6.00)	1 (4.55)	7 (4.64)	3 (14.3)	–
*APOE* ε2/ε4 no. (%)	4 (2.00)	0 (0.00)	2 (1.32)	0 (0.00)	–
*APOE* ε3/ε3 no. (%)	151 (75.5)	11 (50.0)	71 (47.0)	14 (66.7)	–
*APOE* ε3/ε4 no. (%)	33 (16.5)	9 (40.9)	58 (38.4)	4 (19.0)	–
*APOE* ε4/ε4 no. (%)	0 (0.00)	0 (0.00)	13 (8.61)	0 (0.00)	–
*APOE* ε2 carrier (%)	8.00	9.09	5.96	14.3	0.567
*APOE* ε3 carrier (%)	94.0	90.9	86.8	85.7	0.115
*APOE* ε4 carrier (%)	18.5	40.9	48.3	19.0	< 0.0001

Age, education and MMSE scores were analyzed by the Kruskal–Wallis test. Sex ratio and *APOE* alleles carrier frequencies were analyzed using the chi-squared test.

Parameters that were not normally distributed were age in the NC, aMCI, AD, and VaD groups, duration of disease in the aMCI, AD, and VaD groups, age at onset in the AD and VaD groups, education in the NC, AD, and VaD groups and MMSE score in the NC, and AD groups. Age, female-male ratio, education history, MMSE score, and *APOE* ε4 carrier frequency were significantly different among groups (Table 1). No significant differences were found in *APOE* ε2 carrier frequency and *APOE* ε3 carrier frequency among groups (Table 1). A post hoc analysis showed age was significantly higher in the AD and VaD groups compared to the NCs (*P* < 0.0001 and *P* < 0.0001, respectively). The female-male ratio was significantly different as compared with NCs only in the AD group (*P* = 0.018). Education history was significantly lower in the AD and VaD groups as compared with NCs (*P* < 0.0001 and *P* < 0.0001, respectively). MMSE score in the AD and VaD groups were significantly lower as compared with NCs (*P* < 0.0001 and *P* < 0.0001, respectively). Compared with NCs, the *APOE* ε4 carrier frequency was significantly higher in the aMCI and AD groups (*P* = 0.014 and *P* < 0.0001, respectively).

### Blood *COASY* DNA methylation levels

**Figure 3:**
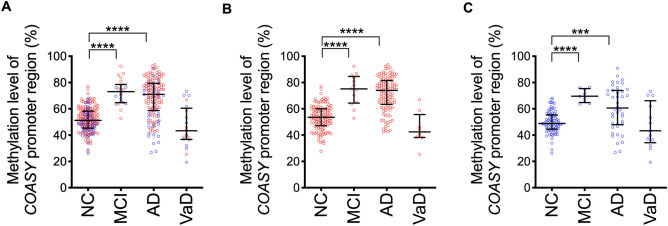
Blood *COASY* DNA methylation levels in dementia patients. This shows blood *COASY* DNA methylation levels in the dementia patients overall (**A**), females only (**B**), and males only (**C**). Red circles indicate females and blue circles males. Horizontal lines indicate medians and error bars interquartile range. *P < 0.05, ***P < 0.001, ****P < 0.0001. Dunn–Bonferroni correction.

*COASY* promoter DNA methylation levels in the AD group did not follow a normal distribution. *COASY* DNA methylation levels were significantly different among groups (Fig. 3A, *P* < 0.0001). A post hoc analysis showed methylation levels in the *COASY* promoter region were higher in the aMCI and AD groups as compared with NCs (Fig. 3A, aMCI, *P* < 0.0001, AD, *P* < 0.0001, VaD, *P* = 0.59).

We investigated the correlations of age, disease duration, age at onset, education years, and MMSE score with *COASY* DNA methylation levels. A significant positive correlation was observed between MMSE score and *COASY* DNA methylation levels in the AD group (Table 2, *P* < 0.0001). No significant correlations were found for the other parameters in any group (Table 2). Therefore, among the background characteristics with significant differences among the groups, age and education history had no influence on *COASY* DNA methylation levels. Further, *COASY* DNA methylation levels were compared by stratifying individuals based on sex and presence of *APOE* ε4. This revealed that *COASY* DNA methylation levels in the NC and AD groups were significantly higher in women [Table 2, NCs: female, 53.1 ± 0.9 (53.6), male, 49.7 ± 0.9 (48.9), *P* = 0.007, AD: female, 71.3 ± 1.2 (74.0), male, 60.4 ± 2.6 (60.6), *P* < 0.0001, mean ± S.E.M. (median)]. No significant differences were found in *COASY* DNA methylation levels between men and women for the other patient groups or due to presence of *APOE* ε4 in any group (Table 2). Further, multiple linear regression analysis found that among age, sex, and MMSE score (factors that could possibly affect *COASY* DNA methylation levels), both MMSE and sex were significantly associated with *COASY* DNA methylation levels in AD (Table 3, *P* < 0.0001, *P* < 0.0001, respectively).

**Table 2:** Associations between individual background characteristics and *COASY* promoter region DNA methylation levels in dementia patients.

*COASY* methylation levels vs	NC (*n* = 200)		MCI (*n* = 22)		AD (*n* = 151)		VaD (*n* = 21)	
	*ρ*	*P*	*ρ*	*P*	*ρ*	*P*	*ρ*	*P*
Age (years)	− 0.10	0.18	0.17	0.45	− 0.14	0.08	0.08	0.74
Sex	–	0.01	–	0.29	–	0.00	–	0.92
Duration of disease (years)	–	–	0.09	0.70	− 0.07	0.43	− 0.36	0.11
Age at onset (years)	–	–	0.14	0.53	− 0.10	0.25	0.20	0.39
Education (years)	− 0.07	0.32	− 0.18	0.43	0.10	0.24	0.16	0.49
MMSE score	0.04	0.56	− 0.30	0.18	0.37	0.00	0.27	0.30
*APOE* ε4 carrier	–	0.45	–	0.70	–	0.44	–	0.32

Associations were examined by Spearman’s rank correlation coefficients, except for sex and *APOE* ε4 carrier. The parameters were compared using the Mann–Whitney U test.

**Table 3:** Multiple linear regression analysis on *COASY* DNA methylation levels in AD.

Variables	*B*	S.E.M	*P*
Age	− 0.12	0.16	0.44
MMSE	0.99	0.18	< 0.0001
Sex	11.01	2.31	< 0.0001

*R*^*2*^ = 0.296, analysis of variance (ANOVA) *P* < 0.0001.

Since we observed a significant difference in sex ratio in the disease groups and a significant difference in *COASY* methylation levels due to sex, we analyzed these data separately for men and women. *COASY* DNA methylation levels were significantly different among groups in both females and males (Fig. 3B,C, *P* < 0.0001 and *P* < 0.0001, respectively). A post hoc analysis revealed that methylation levels in the *COASY* promoter region were higher in the aMCI and AD groups as compared with NCs in both females and males (Fig. 3B,C, Female: aMCI, *P* < 0.0001, AD, *P* < 0.0001, and VaD, *P* = 0.54; Male: aMCI, *P* < 0.001, AD, *P* = 0.0005, and VaD, *P* = 1.00,).

### Receiver operating characteristics (ROC) analysis

**Figure 4:**
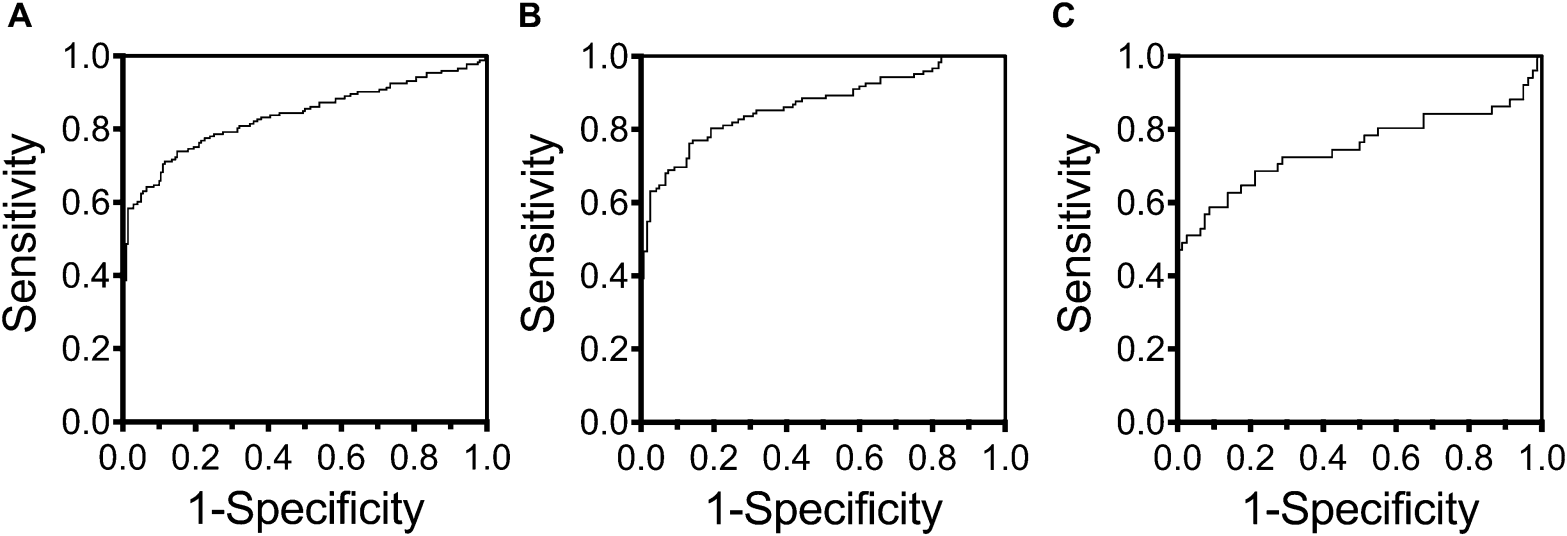
ROC curve for diagnosis in aMCI and AD. This shows the potential sensitivity and specificity of diagnosis for aMCI, AD and all (**A**), females only (**B**), and males only (**C**) using COASY DNA methylation levels.

We carried out ROC analysis to determine whether *COASY* DNA methylation levels could be a useful blood biomarker in the diagnosis of MCI and AD. The results were: area under the curve (AUC): 0.84, 95% Confidence interval (CI): 0.79–0.88 (Fig. 4A). Stratifying the analysis by sex, the result for women was AUC: 0.87, 95% CI: 0.82–0.91 (Fig. 4B) and for men AUC: 0.75, 95% CI: 0.65–0.85 (Fig. 4C).

### Comparison in *COASY* DNA methylation levels in AD without CVD, AD with CVD, and VaD

**Figure 5:**
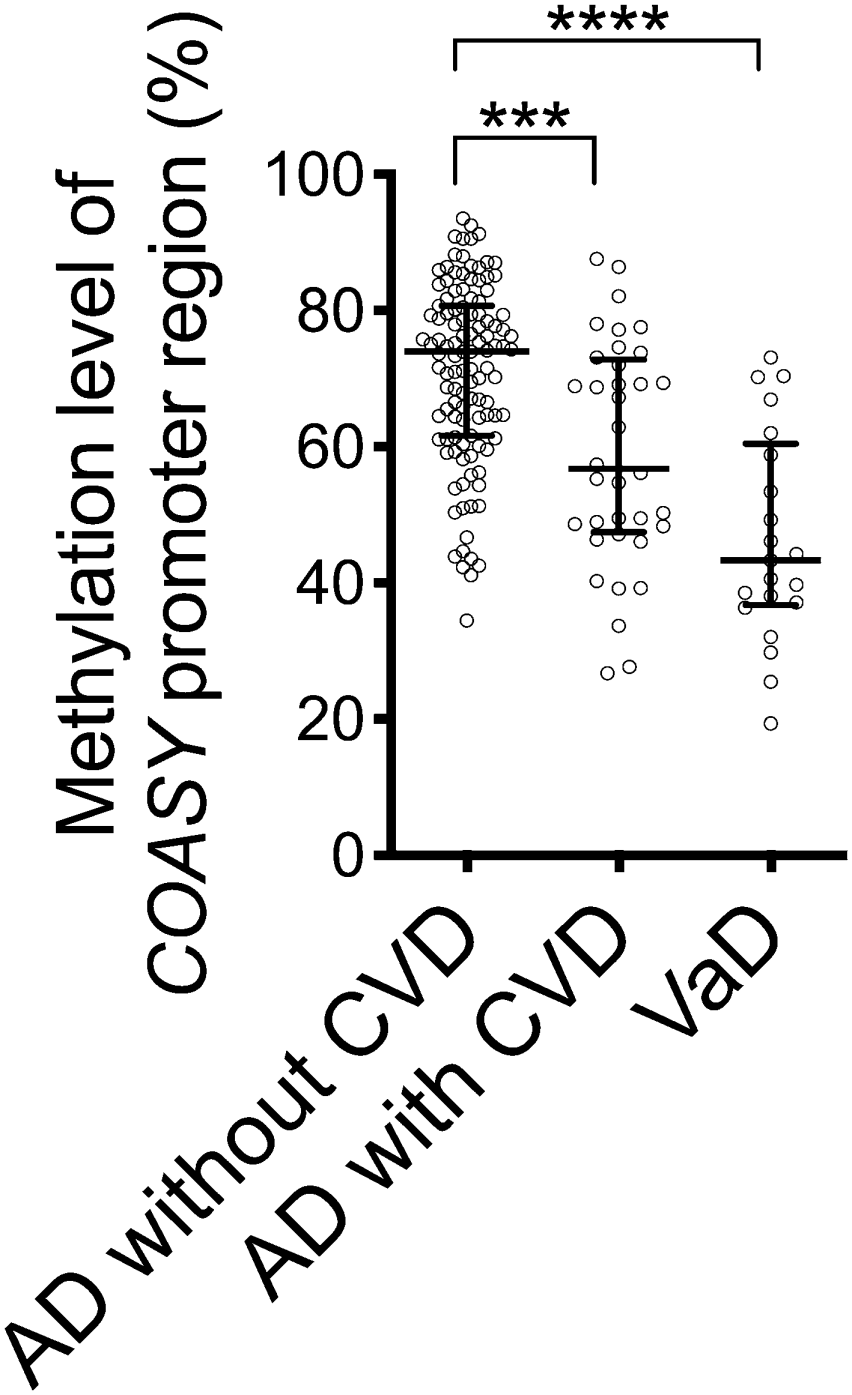
*COASY* promoter region DNA methylation levels in AD without CVD, AD with CVD, and VaD. This shows blood *COASY* DNA methylation levels in AD without CVD, AD with CVD, and VaD. Horizontal lines indicate medians and error bars interquartile range. ***P < 0.001. ****P < 0.0001. Dunn–Bonferroni correction.

We also compared *COASY* promoter DNA methylation levels among the AD without CVD subgroup (*n* = 115), AD with CVD subgroup (*n* = 36), and VaD group (*n* = 21). *COASY* DNA methylation levels were significantly different among these groups (Fig. 5, *P* < 0.0001). *COASY* promoter region DNA methylation levels were significantly higher in the AD without CVD subgroup than in the AD with CVD subgroup and VaD group (Fig. 5, *P* < 0.001 and *P* < 0.0001, respectively). Also, there was a trend-toward tendency for *COASY* promoter region DNA methylation levels to be higher in the AD with CVD subgroup than in the VaD group (Fig. 5, *P* = 0.058).

## Discussion

The present study revealed that blood *COASY* DNA methylation levels were higher in AD and aMCI than those in NCs (Fig. 3A). When comparing the blood *COASY* DNA methylation levels among individuals with AD without CVD, AD with CVD, and VaD, the median value of the methylation levels was highest in individuals with AD without CVD (Fig. 5).

The increased levels of blood *COASY* DNA methylation in AD and aMCI compared to NCs confirmed the reproducibility of our previous findings^13^. Further, in the present study, since the analyses were performed with an increased number of samples, the effect of the sex difference between the NC and AD groups could be assessed (Table 2). In the present study, blood *COASY* DNA methylation was significantly higher in women than in men in both the NC and AD groups (Table 2). Epidemiological research has reported a higher prevalence of AD in women^21^. This may be because the number of women in the elderly population is higher and the effects of hormonal changes are greater in women compared with men; however, the actual reasons remain unclear^22^. In the present study, we considered it possible that the high *COASY* DNA methylation levels in women were associated with the higher prevalence of AD in women. Further, in analysis by sex, compared to NCs, blood *COASY* DNA methylation levels were increased in AD and aMCI in both men and women (Fig. 3B,C). This suggested that *COASY* DNA methylation levels were associated with AD pathology itself, irrespective of sex.

The results of the ROC analysis suggested that, as a blood biomarker, *COASY* DNA methylation levels had adequate potential for use in the diagnosis of MCI and AD (Fig. 4A–C). Application as a clinical diagnostic marker in the early phase of AD onset or at the MCI stage could be foreseen, and in this case, sensitivity and specificity would be expected to increase since *COASY* DNA methylation levels were positively correlated with MMSE score (Table 2).

For VaD, in which cognitive dysfunction is due to cerebrovascular disorders, we observed no significant change in blood *COASY* DNA methylation levels as compared to NCs. When we compared blood *COASY* DNA methylation levels in the AD without CVD and AD with CVD subgroups, they were significantly higher in AD without CVD than AD with CVD and, compared with VaD, there was a tendency for levels to be higher in AD with CVD, although the difference was not significant (Fig. 5). In AD with CVD, although individuals met the clinical diagnosis criteria for AD, vascular elements may have been greatly involved. This suggests that *COASY* DNA methylation levels are higher in AD without CVD in which vascular impairment is minimal.

The above observations suggest that rises in blood *COASY* DNA methylation levels reflect an AD pathology. However, since there was a positive correlation between blood *COASY* DNA methylation levels and MMSE scores (Table 2) and in multiple linear regression analysis with adjustment for age and sex, the effect on MMSE was still observed (Table 3), we considered that, irrespective of the severity of cognitive dysfunction, this reflected neuropathological changes occurring before the appearance of overt symptoms. In addition, in AD, Aβ-plaque accumulation is thought to reach a plateau before the onset of cognitive symptoms^23^. Thus, when AD progresses after symptom appearance in patients and there is a drop in MMSE score, perhaps *COASY* plays an adaptive role.

The *COASY* gene encodes CoA synthase and CoA has very important roles in the human body and the mechanism of acetylcholine esterase inhibitors used to treat AD relies on an increase in acetylcholine in the synaptic cleft, the acetylcholine being synthesized with acetyl-CoA and choline as substrates. While we did not observe a clear change in mRNA levels due to changes in *COASY* DNA methylation levels, our findings suggested that the long-term effect of changes in *COASY* DNA methylation levels would be to cause neurotoxicity. As they were evident before amyloid plaque formation, altered methylation levels could be a useful early blood biomarker.

There are some limitations in the present study. First, the functional roles of CoA synthase and *COASY* DNA methylation in AD onset are unclear. Further studies will be needed to determine if they have a functional role in the cause of neurodegeneration or increases in *COASY* DNA methylation levels are observed in AD when they are used as a surrogate marker. Second, we did not determine the locations where the DNA methylation actually occurred. However, MS-HRM is capable of measuring epigenetic changes^24^. In addition, it is a low-cost method widely used for this purpose. Third, we were unable to investigate an association between Aβ deposition in the brain and *COASY* DNA methylation levels in humans. In the future, it will be necessary to demonstrate this through PET imaging and longitudinal research.

## Conclusion

In the present study, we showed that blood *COASY* DNA methylation levels were increased in individuals with AD and aMCI. Our results suggested that increased *COASY* DNA methylation levels could be a useful biomarker that reflects neurotoxicity in the early stages of AD.
